# Prevalence, Correlates, and Prognosis of Peripheral Artery Disease in Rural Ecuador—Rationale, Protocol, and *Phase I* Results of a Population-Based Survey: An Atahualpa Project-Ancillary Study

**DOI:** 10.1155/2014/643589

**Published:** 2014-09-21

**Authors:** Oscar H. Del Brutto, Mark J. Sedler, Robertino M. Mera, Pablo R. Castillo, Elizabeth H. Cusick, Jadry A. Gruen, Kelsie J. Phelan, Victor J. Del Brutto, Mauricio Zambrano, David L. Brown

**Affiliations:** ^1^School of Medicine, Universidad Espíritu Santo-Ecuador, Guayaquil, Ecuador; ^2^Air Center 3542, P.O. Box 522970, Miami, Fl 33152-2970, USA; ^3^School of Medicine, Stony Brook University, Health Sciences Center, L-4 Room 158, Stony Brook, NY 11794, USA; ^4^Gastroenterology Department, Vanderbilt University, Nashville, TN, USA; ^5^Sleep Disorders Center, Mayo Clinic College of Medicine, 4500 San Pablo Road, Jacksonville, FL 32224, USA; ^6^Community Center, Villa Club Etapa Cosmos Mz 15 Villa 42, Atahualpa, Daule, Guayas, Ecuador; ^7^Cardiovascular Division, Washington University School of Medicine, 660 S. Euclid Avenue, Campus Box 8086, St. Louis, MO 63110, USA

## Abstract

*Background*. Little is known on the prevalence of peripheral artery disease (PAD) in developing countries. *Study design*. Population-based study in Atahualpa. In *Phase I*, the Edinburgh claudication questionnaire (ECQ) was used for detection of suspected symptomatic PAD; persons with a negative ECQ but a pulse pressure ≥65 mmHg were suspected of asymptomatic PAD. In *Phase II*, the ankle-brachial index will be used to test reliability of screening instruments and to determine PAD prevalence. In *Phase III*, participants will be followed up to estimate the relevance of PAD as a predictor of vascular outcomes. *Results*. During *Phase I*, 665 Atahualpa residents aged ≥40 years were enrolled (mean age: 59.5 ± 12.6 years, 58% women). A poor cardiovascular health status was noticed in 464 (70%) persons of which 27 (4%) had a stroke and 14 (2%) had ischemic heart disease. Forty-four subjects (7%) had suspected symptomatic PAD and 170 (26%) had suspected asymptomatic PAD. Individuals with suspected PAD were older, more often women, and had a worse cardiovascular profile than those with nonsuspected PAD. *Conclusions*. Prevalence of suspected PAD in this underserved population is high. Subsequent phases of this study will determine whether prompt detection of PAD is useful to reduce the incidence of catastrophic vascular diseases in the region.

## 1. Introduction

Underserved populations of Latin America are going through a process of epidemiologic transition due to increases in life expectancy and changes in dietary habits and lifestyles [1]. As a consequence, the incidence and prevalence rates of cardiovascular diseases are increasing to the point that these conditions are considered to be the next health epidemics of the region [2]. Epidemiologic surveys assessing specific risk factors are mandatory to define and to respond to the potential burden of ischemic heart disease and stroke in these regions. Such surveys may prove to be cost-effective for developing strategies directed to improve the cardiovascular health of other populations or ethnic groups [3].

Peripheral artery disease (PAD) is the third most common vascular disease, affecting more than 10% of individuals greater than 70 years of age worldwide [4]. This condition is an important marker of systemic atherosclerosis and has been independently associated with an increased risk of ischemic heart disease and stroke [5–7]. Despite this, there is controversy regarding the role of routine screening and prompt detection of PAD [8]. Prior conflicting results have been, at least in part, due to a lack of standardization of diagnostic methods used for PAD diagnosis in population-based surveys, the fact that only either asymptomatic or symptomatic individuals have been screened, and differences in the age, race/ethnicity, and cardiovascular risk status across studied populations [9–11]. Most cohort studies have been conducted in the developed world; little longitudinal information is available from the few cross-sectional surveys assessing PAD prevalence in low- and middle-income countries [12–15]. Thus, we performed a population-based cohort study to determine the prevalence, clinical correlates, evolution, and outcome of PAD in a rural Ecuadorian community, which may be used as a template for other community-based studies attempting to reduce the burden of noncommunicable diseases in rural areas of low- and middle-income countries.

## 2. Methods

### 2.1. Population Studied

Atahualpa is located in rural coastal Ecuador (2°18′S, 80°46′W) and was selected for the study as a village representative of the region. More than 95% of the population belongs to the Native/Mestizo ethnic group (Amerindians). All inhabitants speak Spanish and most men belong to the blue-collar class (artisan carpenters) and most women are homemakers, with a rather homogeneous family income rate that fluctuates from US$ 5,000 to $ 12,000 per year. Atahualpa is relatively isolated and closed; inhabitants do not migrate and many of them have never visited large urban centers. People mobilize within the village mainly by walking or bicycle riding, as very few own a motor vehicle. There are no fast-food restaurants; most people eat at home. The diet is rich in fish and carbohydrates but poor in polyunsaturated fats and dairy products. The village has only one health center of the Minister of Health staffed by general physicians, nurses, dentists, and obstetricians.

### 2.2. The Atahualpa Project

The methodology and operational definitions of the Atahualpa Project have been detailed elsewhere [16–18]. In brief, this multistep, population-based cohort study was designed to reduce the burden of cardiovascular and neurological diseases in the region, by assessing and modifying risk factors through the implementation of intervention strategies directed at informing people about their health status and the best ways to improve it according to specific situations. The main protocols of this project have been registered at http://www.clinicaltrials.gov/ (NCT01627600, NCT01831908, and NCT01877616) and the informed consent forms for all substudies of the Atahualpa Project have been approved by the IRB of Hospital-Clínica Kennedy, Guayaquil-Ecuador (FWA 00006867).

### 2.3. Study Design

This substudy of the Atahualpa Project focused on the evaluation of prevalence, incidence, mortality, and clinical correlates of PAD. It has been divided into three main phases. In* Phase I*, trained field personnel (including rural doctors) screened all Atahualpa residents aged ≥40 years to identify those with suspected PAD. Residents were defined as persons who had been living in the village for at least six months before the start of the survey (15 June, 2013). Persons declining to sign the informed consent were excluded. At this time, cardiovascular risk factors and history of vascular events (stroke and ischemic heart disease) were assessed. During* Phase II*, the ankle-brachial index (ABI) will be used as the gold standard for diagnosis of PAD in order to test the reliability of the screening instruments as well as to determine the actual prevalence of PAD and its clinical correlates. In* Phase III*, all participants will be followed up on a yearly basis to estimate the relevance of PAD as a predictor of vascular outcomes and death.

### 2.4. *Phase I* (Basal Survey)

#### 2.4.1. Detection of Persons with Suspected PAD

We used the Edinburgh claudication questionnaire (ECQ) for detection of cases with suspected symptomatic PAD [19]. This reliable instrument consists of six questions directed not only to detect suspected PAD cases but also to grossly assess their severity. The ECQ was independently translated from its original English version to Spanish by bilingual physicians from our group (O.H.D., R.M.M.). Then, the Spanish version of the ECQ was culturally adapted—including vernacular Spanish words used by local people—with the aid of Atahualpa's community leaders and rural doctors working in the village and tested in a random sample of the population before the study (Table 1). In addition, the pulse pressure was recorded due to its relevance in the evaluation of patients with suspected PAD. It was calculated by subtracting the diastolic pressure from the systolic pressure and a value ≥65 mmHg was considered as suggestive of asymptomatic PAD [20]. On the basis of results from the ECQ and pulse pressure determinations, persons were classified into the following three groups: (1) suspected symptomatic PAD, (2) suspected asymptomatic PAD, and (3) nonsuspected PAD (Table 2).

#### 2.4.2. Identification of Patients with Stroke and Ischemic Heart Disease

To recognize persons with a history of stroke and ischemic heart disease, all Atahualpa residents were screened—during the survey—by rural doctors with the use of validated field questionnaires. The field instrument for the detection of stroke cases was that used for assessment of stroke prevalence in previous surveys performed by our group in the same village [21, 22]. For the detection of cases of ischemic heart disease, we used a validated Spanish translation of the Rose questionnaire [23], and we also asked each person if they have ever had a diagnosis of myocardial infarction. Then, certified neurologists and cardiologists moved to Atahualpa in order to evaluate all suspected cases and a random sample of negative individuals matched by age and sex for each positive case. Stroke was diagnosed, according to the World Health Organization definition, in patients who had experienced a rapidly developing event characterized by clinical signs of focal or global disturbance of cerebral function, lasting ≥24 hours, with no apparent cause other than a vascular cause [24]. Patients were considered to have ischemic heart disease on the basis of clinical judgment or ECG findings [25].

#### 2.4.3. Assessment of Risk Factors

During the survey, demographic characteristics, cardiovascular risk factor, and other PAD correlates of all enrolled persons, such as edentulism, were recorded. Relevant demographic data included age, sex, educational level, and alcohol intake (dichotomized in <50 and ≥50 g per day). Cardiovascular risk factors were assessed in the field by determining the cardiovascular health (CVH) status of participants. For this, we used the seven CVH metrics proposed by the American Heart Association [26]. These included smoking status, body mass index (BMI), physical activity, diet, blood pressure (BP), fasting glucose, and total cholesterol levels. Smoking status and physical activity were based on a self-report; BMI (kg/m^2^) was calculated after obtaining the person's height and weight; diet was assessed by direct interviews with the aid of a validated food frequency questionnaire [27]; BP was measured with a Microlife BP A200 AFIB© digital sphygmomanometer (Microlife Corporation, Taipei, Taiwan), using a well-defined protocol described elsewhere [28]; and fasting glucose and total cholesterol levels were measured by obtaining a capillary blood sample, using Accu-chek Active and Accutrend Plus devices (Roche Diagnostics, Mannheim, Germany), respectively. Each CVH metric was classified as ideal, intermediate, or poor, and the CVH status of a person was classified as poor if at least one CVH metric was in the poor range (Table 3).

Edentulism, which was used as a proxy of periodontitis and chronic inflammation, was also investigated in the entire population, as some studies have shown an association between this covariate and PAD [29, 30]. For this, a rural dentist performed an oral exam during the survey with emphasis on the number of remaining teeth. Individuals were classified in three groups according to whether they had <10, 10–19, or ≥20 teeth.

### 2.5. *Phase II* (Confirmation of Suspected Cases of PAD)

For this phase of the study, trained medical students—under the supervision of a certified cardiologist—will perform the ankle-brachial index (ABI) in all persons who were suspected to have symptomatic (ECQ positive) and asymptomatic PAD (pulse pressure ≥ 65 mm/Hg with a negative ECQ) during* Phase I* and in a random sample of a similar number of negative individuals that will be matched by age and sex with suspected cases. Those with amputations, fractures, or leg ulcers preventing ABI measurements will be excluded.

A manual sphygmomanometer (Welch Allyn Tycos© 7670-01) and a portable vascular Doppler (Nicolet n800©) with an 8 MHz probe will be used for all BP determinations, which will be carried out at the community center of the Atahualpa Project under comfortable temperature levels with the patient resting in the supine position for at least 10 minutes before the test. According to the recommendations of the American Heart Association regarding cuff size and positions, BP will be measured in both arms and legs with the aid of both devices (the sphygmomanometer and the Doppler) following a counterclockwise sequence: right arm, right posterior tibial, right dorsalis pedis, left posterior tibial, left dorsalis pedis, and left arm [31]. ABIs will be reported separately for each leg and calculated by dividing the higher of the posterior tibial or dorsalis pedis BP by the higher right or left arm systolic BP. An ABI ≤ 0.9 will be considered as a diagnostic of lower-limb PAD. Persons with an ABI ≥ 1.4 will not be diagnosed as PAD and will be excluded from the main analysis but will be considered at an increased risk for vascular events and death as these high indices are suggestive of a rigid and incompressible wall in an artery affected by atherosclerosis [32]. Those persons will be followed up and evaluated in a separate analysis.

### 2.6. *Phase III* (Cohort Study)

All participants in the* Phase I* survey will be followed up yearly for at least five years to evaluate PAD progression (defined as a decrease ≥0.15 in the ABI), the rate of transformation of asymptomatic to symptomatic PAD, and the quality of life of symptomatic patients. The latter will be evaluated by the use of the walking impairment questionnaire [33] and the vascular quality of life questionnaire-6 (VascQoL-6), which is an abridged, yet equally reliable, version of the Vasc-QoL-25 [34]. In addition, we will assess the prospective incidence of stroke and ischemic heart disease as well as the mortality rate among persons with and without PAD. For this, we will review death certificates and medical records from the single Health Center of Atahualpa and conduct yearly door-to-door surveys using the same approach as described for* Phase I*. By the end of the study, it will be possible to estimate the relevance of PAD as a predictor of vascular outcomes and death.

### 2.7. Statistical Analyses

All analyses are carried out by using STATA version 13 (College Station, TX, USA). Descriptive statistics are presented as means ± standard deviations for continuous variables and as percentages with 95% CI for categorical variables. A *P* value of less than 0.05 is considered significant. For* Phase I* results, differences on traditional and nontraditional cardiovascular risk factors across persons with and without suspected PAD (symptomatic and asymptomatic) were presented. During* Phase II*, reliability of the ECQ and pulse pressure calculation for detection of patients with symptomatic and asymptomatic PAD will be assessed by calculating their sensitivity and specificity as well as their positive and negative predictive values (using numbers of true and false positive suspected cases and those of true and false negative controls). Using generalized linear analysis, we will evaluate the association between confirmed PAD and all the other variables, after adjusting for age, sex, education, and alcohol intake. Univariate and multivariate analyses will be performed with PAD as the dependent variable; the output of the model will be the odds ratio of a given relationship. To compare the incidence of vascular events and death between those with and without PAD (*Phase III*) we will use time-to-event univariate methods such as Kaplan-Meir curves and the log rank test. Cox proportional regression models (hazard ratios and 95% C.I.) and Poisson regression models (adjusted incidence rates and 95% C.I.) will be used to evaluate progression of PAD and the association between PAD and the incidence of vascular events and death, after adjusting for demographics, cardiovascular risk factors, and other confounders.

## 3. Results (*Phase I*)

The door-to-door census identified 688 Atahualpa residents aged ≥40 years, 23 (3.3%) of whom declined to participate. Mean age of the 665 enrolled persons was 59.5 ± 12.6 years; 384 (58%) were women and 426 (64%) had up to primary school education. Alcohol consumption ≥ 50 g per day was admitted by 114 (17%) persons.

Mean values for the quantitative measures obtained were as follows: height 149 ± 9 cm, weight 60 ± 12 kg, BMI 27 ± 5 kg/m^2^, systolic BP 138 ± 25 mmHg, diastolic BP 77 ± 12 mmHg, pulse pressure 61 ± 21 mmHg, fasting glucose 140 ± 86 mg/dL, and total cholesterol 199 ± 33 mg/dL. In the study cohort, 75 persons (11%) were on antihypertensive, 68 (10%) on hypoglycemic, and 16 (2.4%) on hypocholesterolemic drugs (15% were taking combinations of these drugs).

Overall, 10 persons (1.5%) had all seven CVH metrics in the ideal range (ideal CVH status); 191 (29%) had a combination of ideal and intermediate but not poor CVH metrics (intermediate CVH status); and the remaining 464 (70%) had one or more CVH metrics in the poor range (poor CVH status). Most of these individuals have only one or two CVH metrics in the poor range (mean ± SD1.1 ± 1). The individual CVH metric that was most often in the poor range was BP (36%), followed by fasting glucose levels (30%) and BMI (25%). On the oral exam, 192 (29%) had <10 remaining teeth (severe edentulism). A total of 41 persons (6%) had a history of a vascular event, including stroke in 27 cases and ischemic heart disease in 14 (there were no individuals with history of both stroke and ischemic heart disease).

A total of 205 persons (31%) had a pulse pressure ≥ 65 mmHg. Persons with a high pulse pressure were older, more often women, and less educated than those with a pulse pressure < 65 mmHg. In contrast, alcohol intake ≥50 g per day was most common among persons with normal pulse pressure values. There were no significant differences in mean values of BMI, fasting glucose, and total cholesterol levels across groups of pulse pressure values, but a poor CVH status and severe edentulism were more frequent in those with increased pulse pressure. Also, a history of stroke or ischemic heart disease was more common among persons with an increased pulse pressure (Table 4).

A total of 44 persons (7%) were positive on the ECQ. Thirty-five of them also had increased values of pulse pressure. Only nine of these 44 persons had grade II (severe) suspected intermittent claudication, defined as a positive response to question number 4 (Table 1). Persons with a positive ECQ were older, more frequently women, than those with a negative ECQ. There were no differences in educational levels, alcohol intake, BMI, total cholesterol blood levels, severe edentulism, or history of vascular events across both groups. However, mean fasting glucose levels were higher and the CVH status was more often poor among persons with a positive ECQ (Table 5).

Table 6 summarizes the characteristics of enrolled persons according to whether they were suspected or nonsuspected cases of PAD. There were no differences in demographics or in cardiovascular risk factors across categories of suspected PAD (symptomatic or asymptomatic). In contrast, with the exception of BMI, diastolic BP, and total cholesterol blood levels, persons with nonsuspected PAD differed significantly from those with either symptomatic or asymptomatic PAD.

## 4. Discussion

It has been estimated that more than 200 million individuals worldwide are afflicted with PAD [4]. The disease has been described as a pandemic sparing no nation [35]. The current study confirms a high prevalence of suspected PAD in the adult population of a rural village located in coastal Ecuador. Forty-four persons (7%) had suspected symptomatic PAD and 170 (26%) had suspected asymptomatic PAD. While these numbers have to be confirmed during* Phase II* of this study, they are consistent with the high stroke prevalence (31‰) and with the high number of fatal ischemic heart disease cases that we recently found in Atahualpa [22, 36]. Of note, these findings support previous estimates that the incidence of cardiovascular diseases is increasing in underserved populations of Latin America [2]. Consistent with previous studies [37], individuals with suspected PAD were more often women and had more modifiable cardiovascular risk factors than those without suspected PAD.

Published data on PAD from Latin America are scarce. A recent comprehensive review identified 12 population-based studies conducted in low- and middle-income countries of which only two came from urban centers in Latin America and none were from rural areas [4]. In the Brazilian study (1,008 subjects aged ≥30 years), the prevalence of PAD was 20% [38] while in the Mexican study (400 subjects aged ≥ 40 years) it was 10% [39]. In both studies, PAD was associated with more cardiovascular risk factors; however, both studies were cross-sectional and no data on the incidence of vascular events was available. Our cohort study will fill a gap in the literature by proving unbiased data on PAD prevalence, clinical correlates, progression, and related vascular outcomes in community-dwelling adults living in a rural South American village. The methodology and operational definitions used in this Atahualpa Project-ancillary study could be applied in other population-based studies conducted in rural areas of other middle- and low-income countries to help health authorities to implement strategies directed to reduce the burden of cardiovascular diseases in the region.

The rigorous methodology and comprehensive inclusion of residents of an isolated community in this study will provide a unique opportunity to estimate the prevalence of PAD and its risk factors. However, we will use the ABI to confirm the diagnosis and not physiologic methods of PAD detection. Therefore, it is possible that the true prevalence of PAD might be underestimated; this is a limitation of the current study. Another potential limitation could be the relatively small sample size, which may create some problems in the multivariate adjusted model that will be used during* Phase II* and* Phase III* of this study.

In summary, PAD appears to be highly prevalent in this rural population in Ecuador confirming the global nature of the PAD pandemic and its predilection for persons of both sexes with modifiable cardiovascular risk factors in low- and middle-income countries.

## Figures and Tables

**Table 1:** Culturally adapted Spanish translation of the Edinburgh claudication questionnaire [*with original English version*] used in Atahualpa residents.

(1) Usted siente dolor o una sensación desagradable en una o ambas piernas cuando camina? [*Do you get a pain or discomfort in your leg(s) when you walk?*]	
□_1_ Si [*yes*]	
□_2_ No [*no*]	
□_3_ No puedo caminar [*I am unable to walk*]	
**Si usted contestó que “si” a la pregunta 1, por favor responda las siguientes preguntas. En caso contrario, no hay necesidad de seguir adelante. [*If you answered “yes” to question (1), please answer the following questions. Otherwise you need not continue.*]**	
(2) Este dolor a veces comienza cuando usted se encuentra quieto, de pie o sentado? [*Does this pain ever begin when you are standing still or sitting?*]	
□_1_ Si [*yes*]	
□_2_ No [*no*]	
(3) Este dolor se presenta cuando usted camina cuesta arriba o de prisa? [*Do you get it if you walk uphill or hurry?*]	
□_1_ Si [*yes*]	
□_2_ No [*no*]	
(4) Este dolor se presenta cuando usted camina a paso normal a nivel de la tierra? [*Do you get it when you walk at an ordinary pace on the level?*]	
□_1_ Si [*yes*]	
□_2_ No [*no*]	
(5) Que sucede con este dolor si usted deja de caminar y se queda parado? [*What happens to it if you stand still?*]	
□_1_ Suele persistir por más de 10 minutos [*Usually continues more than 10 minutes*]	
□_2_ Suele desaparecer en 10 minutos o menos [*Usually disappears in 10 minutes or less*]	
(6) Señale con una “x” en este dibujo en que parte (de las piernas) siente usted el dolor o la sensación desagradable [*Where do you get this pain or discomfort? Mark the place(s) with “x” on the diagram below*]	

**Table 2:** Operational categories of PAD suspicion used in the Atahualpa Project.

*Suspected Symptomatic PAD *	
(i) Positive Edinburgh claudication questionnaire and increased pulse pressure∗	
(ii) Positive Edinburgh claudication questionnaire and normal pulse pressure	
*Suspected Asymptomatic PAD *	
Negative Edinburgh claudication questionnaire and increased pulse pressure∗	
*Nonsuspected PAD *	
Negative Edinburgh claudication questionnaire and normal pulse pressure	

^*^Defined as ≥65 mmHg.

**Table 3:** Cardiovascular health metrics and status according to the American Heart Association.

Cardiovascular Health Metrics	
(1) Smoking: ideal (never or quit >1 year), intermediate (quit ≤1 year), and poor (current smoker).	
(2) Body mass index: ideal (<25 kg/m^2^), intermediate (25 to <30 kg/m^2^), and poor (≥30 kg/m^2^).	
(3) Physical activity: ideal (≥150 minutes/week moderate intensity or ≥75 minutes/week vigorous intensity or equivalent combination), intermediate (1–149 minutes/week moderate intensity or 1–74 minutes/week vigorous intensity or equivalent combination), and poor (no moderate and vigorous activity).	
(4) Diet: ideal (4-5 healthy components), intermediate (2-3 healthy components), and poor (0-1 healthy component); based on 5 health dietary components (≥4.5 cups fruits and vegetables/day, ≥two 3.5-oz servings fish/week, ≥three 1-oz equivalent servings fiber-rich whole grains/day, <1,500 mg sodium/day, and ≤450 kcal sugar-sweetened beverages/week).	
(5) Total cholesterol: ideal (untreated and <200 mg/dL), intermediate (treated to <200 mg/dL or 200–239 mg/dL), and poor (≥240 mg/dL).	
(6) Blood pressure: ideal (untreated and <120/<80 mmHg), intermediate (treated to <120/<80 mmHg or 120–139/80–89 mmHg), and poor (≥140/90 mmHg).	
(7) Fasting glucose: ideal (untreated and <100 mg/dL), intermediate (treated to <100 mg/dL or 100–125/mg/dL), and poor (≥126 mg/dL).	
Cardiovascular Health Status	
(1) Ideal CVH status: all seven CVH metrics in the ideal range.	
(2) Intermediate CVH status: CVH metrics in the ideal and intermediate range, but no poor metrics.	
(3) Poor CVH status: at least one CVH metric in the poor range.	

**Table 4:** Characteristics of Atahualpa residents aged ≥40 years according to pulse pressure levels.

	Total series *n* = 665	Pulse pressure (mmHg)		*P* value
		≥65 (*n* = 205)	<65 (*n* = 460)	
Age (mean ± SD)	59.5 ± 12.6	68.4 ± 11.7	55.6 ± 10.9	0.0001
Women, *n* (%)	384 (58%)	134 (65%)	250 (54%)	0.008
Up to primary school, *n* (%)	426 (64%)	157 (77%)	269 (58%)	0.0001
Alcohol intake ≥50 g/day, *n* (%)	114 (17%)	23 (11%)	91 (20%)	0.007
Current smokers, *n* (%)	12 (2%)	3 (1.5%)	9 (2%)	0.659
Body mass index, kg/m^2^ (mean ± SD)	27 ± 5	27 ± 5	27 ± 5	*⋯*
Fasting glucose, mg/dL (mean ± SD)	140 ± 86	142 ± 92	135 ± 83	0.332
Total cholesterol, mg/dL (mean ± SD)	199 ± 33	198 ± 32	199 ± 33	0.716
Poor CVH status, *n* (%)	464 (70%)	187 (91%)	277 (60%)	0.0001
Severe edentulism, *n* (%)	192 (29%)	85 (41%)	107 (23%)	0.0001
Stroke or ischemic heart disease, *n* (%)	41 (6%)	25 (12%)	16 (3%)	0.0001

**Table 5:** Characteristics of Atahualpa residents aged ≥40 years according to the Edinburgh claudication questionnaire.

	Total series *n* = 665	Edinburgh claudication questionnaire		*P* value
		positive (*n* = 44)	negative (*n* = 621)	
Age (mean ± SD)	59.5 ± 12.6	66.3 ± 11.9	59 ± 12.5	0.0001
Women, *n* (%)	384 (58%)	32 (73%)	352 (57%)	0.054
Up to primary school, *n* (%)	426 (64%)	30 (68%)	396 (64%)	0.67
Alcohol intake ≥50 g/day, *n* (%)	114 (17%)	5 (11%)	109 (18%)	0.396
Current smokers, *n* (%)	12 (2%)	1 (2.3%)	11 (1.8%)	0.809
Body mass index, kg/m^2^ (mean ± SD)	27 ± 5	27 ± 5	27 ± 5	*⋯*
Fasting glucose, mg/dL (mean ± SD)	140 ± 86	173 ± 105	137 ± 84	0.007
Total cholesterol, mg/dL (mean ± SD)	199 ± 33	200 ± 30	199 ± 33	0.845
Poor CVH status, *n* (%)	464 (70%)	40 (91%)	424 (68%)	0.003
Severe edentulism, *n* (%)	192 (29%)	16 (36%)	176 (28%)	0.335
Stroke or ischemic heart disease, *n* (%)	41 (6%)	4 (9%)	37 (6%)	0.610

**Table 6:** Characteristics of Atahualpa residents aged ≥40 years according to the categories of peripheral artery disease (PAD) suspicion.

	Total series (*n* = 665)	Suspected symptomatic PAD (*n* = 44)∗	Suspected asymptomatic PAD (*n* = 170)^§^	Nonsuspected PAD (*n* = 451)^‡^	*P* value
Age (mean ± SD)	59.5 ± 12.6	66.3 ± 11.9	68.1 ± 12	55.6 ± 10.9	0.0001
Women, *n* (%)	384 (58%)	32 (73%)	108 (64%)	244 (54%)	0.015
Up to primary school, *n* (%)	426 (64%)	30 (68%)	134 (79%)	262 (58%)	0.0001
Alcohol intake ≥50 g/day, *n* (%)	114 (17%)	5 (11%)	19 (11%)	90 (20%)	0.02
Current smokers, *n* (%)	12 (2%)	1 (2.3%)	2 (1.2%)	9 (2%)	0.767
Body mass index, kg/m^2^ (mean ± SD)	27 ± 5	27 ± 5	27 ± 5	27 ± 5	*⋯*
Systolic BP, mmHg (mean ± SD)	138 ± 25	158 ± 26	164 ± 27	126 ± 13	0.0001
Diastolic BP, mmHg (mean ± SD)	77 ± 12	78 ± 13	79 ± 15	77 ± 10	0.159
BP ≥140/90 mmHg, *n* (%)	242 (36%)	32 (73%)	138 (81%)	72 (16%)	0.0001
Fasting glucose, mg/dL (mean ± SD)	140 ± 86	173 ± 105	148 ± 91	133 ± 81	0.004
Fasting glucose ≥126 mg/dL, *n* (%)	197 (30%)	21 (48%)	59 (35%)	117 (26%)	0.003
Total cholesterol, mg/dL (mean ± SD)	199 ± 33	200 ± 30	198 ± 32	199 ± 33	0.914
Poor CVH status, *n* (%)	464 (70%)	40 (91%)	155 (91%)	269 (60%)	0.0001
Severe edentulism, *n* (%)	192 (29%)	16 (36%)	72 (42%)	104 (23%)	0.0001
Stroke or ischemic heart disease, *n* (%)	41 (6%)	4 (9%)	21 (12%)	16 (4%)	0.0002

^*^Positive Edinburgh claudication questionnaire irrespective of pulse pressure levels; ^§^increased pulse pressure levels and a negative Edinburgh claudication questionnaire; ^‡^normal pulse pressure levels and a negative Edinburgh claudication questionnaire.
